# Complicated versus uncomplicated acute appendicitis: clinical characteristics, diagnostic scores, surgical management, and outcomes in a tertiary hospital in Mogadishu, Somalia

**DOI:** 10.3389/fsurg.2026.1871490

**Published:** 2026-07-27

**Authors:** Abdisalam Ismail Hassan, Shu’ayb Mohamed Hassan, Yakub Burhan Abdullahi, Abdikani Mohamednor Mohamed, Nimo Mohamednor Mohamed, Nuradin Mohamed Nur, Balqiz Mohamed Kulle, Abdijalil Abdullahi Ali, Ahmed Muhammad Bashir

**Affiliations:** 1 Faculty of Medicine and Health Sciences, Jamhuriya University of Science and Technology, Mogadishu, Somalia; 2 Department of Internal Medicine, Somali Sudanese Specialized Hospital, Mogadishu, Somalia; 3 Department of Research and Education, Mogadishu Virtual Academy (MOVA), Mogadishu, Somalia; 4 Center for Health Research and Innovation, Somali National University, Mogadishu, Somalia; 5 Department of General Surgery, Mogadishu Somali–Türkiye Recep Tayyip Erdoğan Training and Research Hospital, Mogadishu, Somalia; 6 Department of Anesthesia and Critical Care, Mogadishu Somali–Türkiye Recep Tayyip Erdoğan Training and Research Hospital, Mogadishu, Somalia; 7 Department of Cardiovascular Surgery, Mogadishu Somali–Türkiye Recep Tayyip Erdoğan Training and Research Hospital, Mogadishu, Somalia

**Keywords:** AIR score, Alvarado score, complicated appendicitis, diagnostic scoring systems, laparoscopic appendectomy, resource-limited settings, RIPASA score, surgical outcomes

## Abstract

**Background:**

Acute appendicitis is the leading cause of acute surgical abdomen worldwide and is associated with substantial morbidity, particularly in complicated cases. Differentiating complicated from uncomplicated appendicitis remains challenging, especially in resource-limited settings where delayed presentation is common. This study compared the clinical characteristics, diagnostic scores, surgical management, and outcomes of complicated and uncomplicated acute appendicitis in a tertiary hospital in Mogadishu, Somalia.

**Methods:**

A retrospective cohort study was conducted among 168 patients who underwent appendectomy between January and December 2025. Data were extracted from medical records and analyzed using R software. Multivariable logistic regression was performed to identify independent predictors of complicated appendicitis.

**Results:**

Complicated appendicitis accounted for 36.3% of cases. Delayed presentation beyond 48 h was more common in complicated appendicitis but was not independently associated with disease severity after multivariable adjustment. Patients with complicated appendicitis had significantly higher inflammatory markers and higher median Alvarado and Appendicitis Inflammatory Response (AIR) scores, whereas modified Alvarado and RIPASA scores did not differ significantly. Because formal diagnostic performance analyses were not performed, these findings should be interpreted as descriptive comparisons rather than evidence of superior diagnostic accuracy. Laparoscopic appendectomy was the predominant surgical approach, although open surgery and conversion were more frequent in complicated cases. Complicated appendicitis was also associated with higher rates of intra-abdominal complications, ICU admission, and longer hospital stay. Increasing age (adjusted OR 1.03, 95% CI 1.00–1.07) and elevated C-reactive protein (adjusted OR 1.11 per 10 mg/L increase, 95% CI 1.05–1.17) were independently associated with complicated appendicitis.

**Conclusion:**

Complicated appendicitis was associated with worse postoperative outcomes. Increasing age and elevated C-reactive protein were independent predictors of complicated appendicitis, whereas delayed presentation was not independently associated after adjustment. Higher median Alvarado and AIR scores were observed in complicated appendicitis; however, formal diagnostic accuracy analyses were not performed. Prospective studies are needed to validate risk stratification tools and improve early diagnosis and timely surgical management in resource-limited settings.

## Introduction

Acute appendicitis is the leading cause of acute surgical abdomen worldwide and remains one of the most common indications for emergency surgery across all age groups (1, 2). It carries a lifetime risk of approximately 7%–8%, representing a substantial global health burden, with an estimated incidence ranging from 86.2 to 106 cases per 100,000 population (1, 2). Although historically more prevalent in Western countries, its incidence has been steadily increasing in developing regions, reflecting evolving epidemiological trends and healthcare dynamics (2).

Clinically, acute appendicitis is broadly classified into uncomplicated and complicated forms. Complicated appendicitis encompasses advanced disease states characterized by perforation, gangrene, peri-appendicular abscess, phlegmon, or localized and generalized peritonitis, whereas uncomplicated appendicitis refers to an inflamed appendix without these features (3–7).

Despite its frequency, acute appendicitis is often underestimated, and a substantial proportion of patients present with advanced disease. Of the approximately 300,000 appendectomies performed annually, nearly one-quarter are attributed to complicated appendicitis (8, 9). The reported proportion varies widely and may reach up to 50% in certain populations, particularly in resource-limited settings (8, 10).

The diagnosis of complicated appendicitis is typically established intraoperatively based on macroscopic findings and confirmed by histopathological examination. If not managed promptly, acute appendicitis may progress to necrosis, gangrene, and perforation. While uncomplicated appendicitis is primarily treated surgically, complicated cases often require additional interventions, including antibiotic therapy, percutaneous drainage, and, in selected cases, delayed appendectomy (8, 9). Compared with uncomplicated disease, complicated appendicitis is associated with significantly worse outcomes, including prolonged hospitalization, increased use of broad-spectrum antibiotics, and higher morbidity (8, 11).

In Somalia, there is a paucity of comprehensive data examining the clinical characteristics, diagnostic approaches, and outcomes of acute appendicitis, particularly in differentiating between complicated and uncomplicated forms. Given the challenges of delayed presentation and limited healthcare resources, identifying factors associated with complicated appendicitis is essential for improving early diagnosis, optimizing management, and reducing morbidity.

Therefore, this study aimed to compare the clinical characteristics, diagnostic scores, surgical management, and outcomes between patients with complicated and uncomplicated acute appendicitis at a tertiary hospital in Mogadishu, Somalia. Additionally, the study sought to identify factors associated with complicated appendicitis to support early risk stratification and guide timely surgical intervention. To our knowledge, this is among the first comprehensive studies from Somalia evaluating clinical characteristics, diagnostic scoring systems, surgical management, and outcomes of complicated versus uncomplicated acute appendicitis.

## Materials and methods

### Study design and setting

This was a hospital-based retrospective cohort study conducted at Mogadishu Somali–Türkiye Recep Tayyip Erdoğan Training and Research Hospital, a major tertiary referral and teaching hospital located in Mogadishu, Somalia. The hospital has a capacity of approximately 250 inpatient beds, 7 operating rooms, and 28 adult intensive care unit (ICU) beds, in addition to a neonatal ICU (14 beds) and a pediatric ICU (10 beds).

The facility is equipped with advanced diagnostic and therapeutic services, including digital radiography, ultrasonography, computed tomography (CT), 1.5 Tesla magnetic resonance imaging (MRI), fluoroscopy, and interventional radiology services such as ultrasound- and CT-guided procedures. As a national referral center, it serves patients from across Somalia and provides residency training in multiple medical specialties.

The study period spanned one year, from January 1, 2025, to December 31, 2025. All eligible patients during this period were included. Although a formal sample size calculation was not performed due to the retrospective design, the sample size was considered adequate based on the total number of cases available during the study period and consistency with similar studies conducted in comparable settings.

### Study population

The study included all adult patients diagnosed with acute appendicitis who underwent surgical management at the study hospital during the study period. Patients aged 18 years and older who underwent appendectomy, either open or laparoscopic, were eligible for inclusion.

Only patients with complete medical records, including clinical presentation, laboratory findings, intraoperative details, and outcome data, were included. Patients managed non-operatively, those who underwent surgery at other institutions and were subsequently referred, and those with alternative intraoperative diagnoses were excluded. Additionally, patients with incomplete or missing key data, including operative findings, essential laboratory results, or outcome status, were excluded.

Restricting the analysis to surgically managed patients may preferentially include individuals with more advanced disease, thereby introducing potential selection bias and limiting generalizability.

### Data collection procedures

Data were collected retrospectively from patient medical records, operative notes, and hospital electronic databases using a structured and pre-tested data abstraction form.

The following variables were collected:

- Sociodemographic data: age and sex
- Clinical presentation: right lower quadrant pain, nausea, vomiting, fever, and duration of symptoms
- Laboratory parameters: white blood cell count, C-reactive protein (CRP), hemoglobin, and neutrophil percentage
- Diagnostic approach: imaging modalities (ultrasound, CT, or both)
- Clinical scoring systems: Alvarado score, modified Alvarado score, Appendicitis Inflammatory Response (AIR) score, and RIPASA score
- Intraoperative findings: appendiceal condition and presence of perforation, abscess, or peritonitis
- Treatment characteristics: surgical approach (open or laparoscopic)
- Outcomes: postoperative complications, ICU admission, and length of hospital stay

Data quality was ensured through the use of a standardized data collection tool, training of data collectors, and routine supervision with cross-checking for completeness and accuracy. Double data entry and validation procedures were performed to minimize data entry errors.

### Handling of missing data

**Figure 1:**
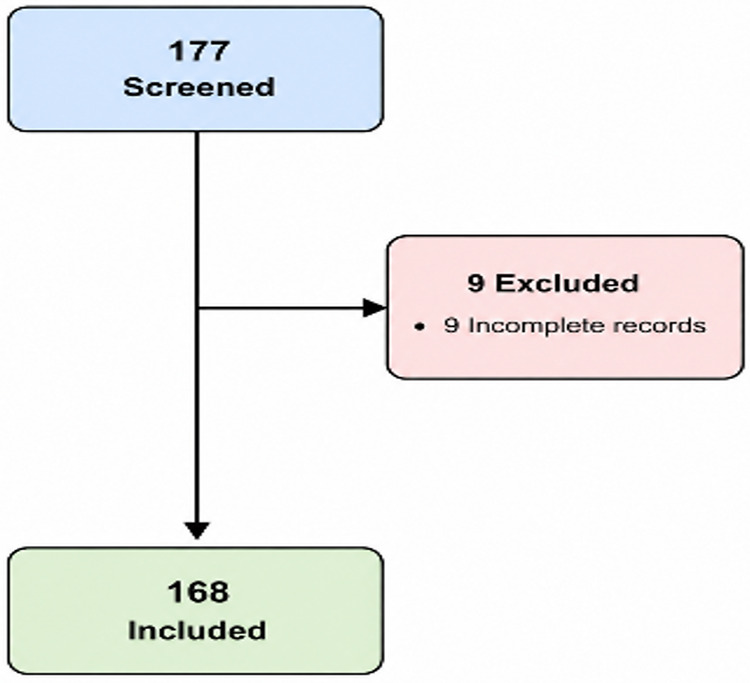
Study flow diagram.

Patients with missing key variables required for study eligibility were excluded before analysis according to the predefined exclusion criteria. For the included study population, the proportion of missing data for all analyzed variables was less than 5%. Given the minimal extent of missingness, complete-case analysis was performed without imputation, as the small proportion of missing data was unlikely to materially affect the study findings. A patient selection flow diagram Figure 1) illustrates the screening process, exclusions, and final study population.

### Operational definitions

Acute appendicitis was classified intraoperatively into:

- Complicated appendicitis: presence of perforation, gangrene, peri-appendicular abscess, or localized/generalized peritonitis
- Uncomplicated appendicitis: inflamed appendix without perforation, gangrene, or abscess

This classification was based on intraoperative findings in accordance with widely accepted surgical definitions.

- Surgical site infection (SSI) was defined according to the Centers for Disease Control and Prevention (CDC) criteria.
- Intra-abdominal abscess was defined as a postoperative intra-abdominal collection confirmed radiologically or during re-intervention.
- Postoperative ileus was defined as delayed return of bowel function requiring prolonged hospitalization or additional supportive treatment.
- Length of hospital stay was calculated from admission to discharge.
- Postoperative complications were assessed during the index hospitalization.
- ICU admission was defined as postoperative admission to the intensive care unit for monitoring or management of severe clinical conditions.

### Data analysis

Statistical analysis was performed using R statistical software (version 4.4.0).

Continuous variables were assessed for normality using histograms and the Shapiro–Wilk test. As most variables were not normally distributed, they were summarized using medians and interquartile ranges (IQR). Categorical variables were presented as frequencies and percentages.

Comparisons between complicated and uncomplicated appendicitis were conducted using appropriate statistical tests. The Wilcoxon rank-sum (Mann–Whitney U) test was used for continuous variables, while the chi-square test or Fisher's exact test was applied for categorical variables, as appropriate.

Candidate variables considered for multivariable logistic regression included age, sex, symptom duration, vomiting, fever, white blood cell count, and C-reactive protein. Variables with *p* < 0.20 in univariable analysis and variables considered clinically relevant based on previous literature were entered into the multivariable model. Diagnostic scores and intraoperative findings were not included because they either reflected disease severity directly or were components of the outcome definition.

Adjusted odds ratios (ORs) with 95% confidence intervals (CIs) were reported. A two-sided *p*-value of <0.05 was considered statistically significant.

### Ethical considerations

Ethical approval was obtained from the Institutional Review Board of Mogadishu Somali–Türkiye Recep Tayyip Erdoğan Training and Research Hospital (Reference No: MSTH/19885).

Due to the retrospective nature of the study and the use of anonymized patient data, the Institutional Review Board waived the requirement for informed consent. All procedures complied with the Declaration of Helsinki and institutional ethical standards.

## Results

### Baseline clinical and laboratory characteristics

A total of 168 patients with acute appendicitis were included, comprising 61 (36.3%) with complicated appendicitis and 107 (63.7%) with uncomplicated appendicitis.

Patients with complicated appendicitis were older, with a median age of 31.0 years (IQR: 25.0–40.0), compared to 28.0 years (IQR: 23.0–34.0) in the uncomplicated group. Male predominance was observed in both groups, with no statistically significant difference in sex distribution.

Right lower quadrant abdominal pain was the most common presenting symptom in both groups (96.7% vs. 97.2%). Other clinical features, including nausea, vomiting, anorexia, fever, diarrhea, dysuria, constipation, abdominal tenderness, and epigastric pain, were similarly distributed, with no statistically significant differences between groups.

A significant difference was observed in symptom duration. Patients with complicated appendicitis were less likely to present within 48 h (70.5% vs. 90.7%) and more likely to present after 48 h (29.5% vs. 10.3%). This association was statistically significant in the unadjusted comparison.

Laboratory findings demonstrated significantly higher inflammatory markers in the complicated group. Median white blood cell count was elevated (12.4 vs. 11.0 × 10^9^/L), and C-reactive protein (CRP) levels were markedly higher (140.0 vs. 33.5 mg/L), indicating a more pronounced systemic inflammatory response. Hemoglobin levels and neutrophil percentages did not differ significantly between groups.

All baseline clinical and laboratory characteristics are summarized in Table 1.

**Table 1:** Baseline clinical and laboratory characteristics of patients with complicated and uncomplicated acute appendicitis.

Characteristic	Complicated appendicitis (*n* = 61)	Uncomplicated appendicitis (*n* = 107)	*p*-value
Age, years	31.0 (25.0, 40.0)	28.0 (23.0, 34.0)	0.056
Sex			0.90
Male	43 (70.5)	74 (69.2)	
Female	18 (29.5)	33 (30.8)	
Right lower quadrant pain	59 (96.7)	104 (97.2)	0.60
Nausea	21 (34.4)	39 (36.4)	0.80
Vomiting	38 (62.3)	63 (58.9)	0.70
Anorexia	3 (4.9)	8 (7.5)	0.70
Fever	12 (19.7)	18 (16.8)	0.50
Diarrhea	2 (3.3)	2 (1.9)	0.60
Dysuria	0 (0.0)	1 (0.9)	>0.99
Constipation	2 (3.3)	4 (3.7)	>0.99
Abdominal tenderness	15 (24.6)	30 (28.0)	0.60
Epigastric pain	2 (3.3)	5 (4.7)	>0.99
Symptom duration <48 h	43 (70.5)	97 (90.7)	<0.001
Symptom duration >48 h	18 (29.5)	11 (10.3)	0.002
White blood cell count	12.4 (10.0, 15.0)	11.0 (7.8, 13.9)	0.013
C-reactive protein	140.0 (42.0, 215.0)	33.5 (12.5, 69.5)	<0.001
Hemoglobin	13.5 (11.6, 15.4)	14.0 (12.8, 15.1)	0.30
Neutrophils, %	78.8 (65.1, 89.1)	80.2 (66.2, 86.1)	>0.99

IQR, interquartile range; CRP, C-reactive protein.

Data are presented as median (IQR) or *n* (%). *P*-values were obtained using the Wilcoxon rank-sum test for continuous variables and chi-square or Fisher's exact test for categorical variables, as appropriate.

### Diagnostic approaches and treatment characteristics

The use of imaging modalities did not differ significantly between groups, with combined ultrasonography and computed tomography being the most frequently utilized diagnostic approach.

The appendix was most commonly located in a retrocecal position in both groups (93.4% vs. 85.0%), without a statistically significant difference.

Patients with complicated appendicitis had significantly higher median Alvarado and Appendicitis Inflammatory Response (AIR) scores than those with uncomplicated appendicitis (both median 7.0 vs. 6.0). In contrast, no statistically significant differences were observed for the modified Alvarado and RIPASA scores. Because this study compared score distributions between groups rather than formally evaluating diagnostic accuracy, these findings should not be interpreted as evidence of superior diagnostic performance.

Regarding surgical management, laparoscopic appendectomy was the predominant approach overall, particularly in uncomplicated cases (95.3% vs. 73.8%). Open appendectomy (19.7% vs. 2.8%) and conversion from laparoscopic to open surgery (6.6% vs. 0.9%) were significantly more frequent among patients with complicated appendicitis, reflecting increased technical complexity in advanced disease.

Detailed diagnostic approaches and treatment characteristics are presented in Table 2.

**Table 2:** Diagnostic approaches and treatment characteristics by appendicitis severity.

Characteristic	Complicated appendicitis (*n* = 61)	Uncomplicated appendicitis (*n* = 107)	*p*-value
Imaging modality			0.11
Both USG and CT	35 (57.4)	55 (51.4)	
CT only	13 (21.3)	22 (20.6)	
No imaging	6 (9.8)	4 (3.7)	
USG only	7 (11.5)	26 (24.3)	
Appendix position			0.30
Retrocecal	57 (93.4)	91 (85.0)	
Pelvic	0 (0.0)	4 (3.7)	
Pre-ileal	1 (1.6)	6 (5.6)	
Post-ileal	1 (1.6)	2 (1.9)	
Paracecal	1 (1.6)	1 (0.9)	
Subhepatic	1 (1.6)	0 (0.0)	
Subcecal	0 (0.0)	3 (2.8)	
Alvarado score	7.0 (6.0–7.0)	6.0 (4.0–7.0)	0.007
Modified Alvarado score	5.0 (4.0–7.0)	5.0 (4.0–7.0)	0.40
AIR score	7.0 (6.0–8.0)	6.0 (4.0–7.0)	0.005
RIPASA score	7.0 (5.5–8.0)	6.0 (5.5–8.0)	0.50
Surgical approach			<0.001
Converted	4 (6.6)	1 (0.9)	
Laparoscopic	45 (73.8)	102 (95.3)	
Open	12 (19.7)	3 (2.8)	

Data are presented as *n* (%) or median (IQR).

*P*-values were calculated using Fisher's exact test for categorical variables and the Wilcoxon rank-sum test for continuous variables.

### Intraoperative findings, histopathology, and postoperative outcomes

As expected, perforation, gangrene, appendicular abscess, and localized or generalized peritonitis were observed exclusively or predominantly among patients classified as having complicated appendicitis because these findings constitute the operational definition of the outcome. These variables are presented to describe the intraoperative characteristics of the two groups and should not be interpreted as independent predictors or inferential comparisons. Conversely, simple appendicitis predominated in the uncomplicated appendicitis group (86.0% vs. 11.5%).

Histopathological findings were consistent with intraoperative observations, with gangrenous appendicitis more frequently identified in the complicated group, while rates of acute and suppurative appendicitis were comparable between groups.

Postoperative outcomes were significantly worse in patients with complicated appendicitis. The incidence of intra-abdominal abscess was higher (9.8% vs. 0.9%), as was ICU admission (8.2% vs. 0.0%). Surgical site infection occurred more frequently in the complicated group (6.6% vs. 0.9%).

**Figure 2:**
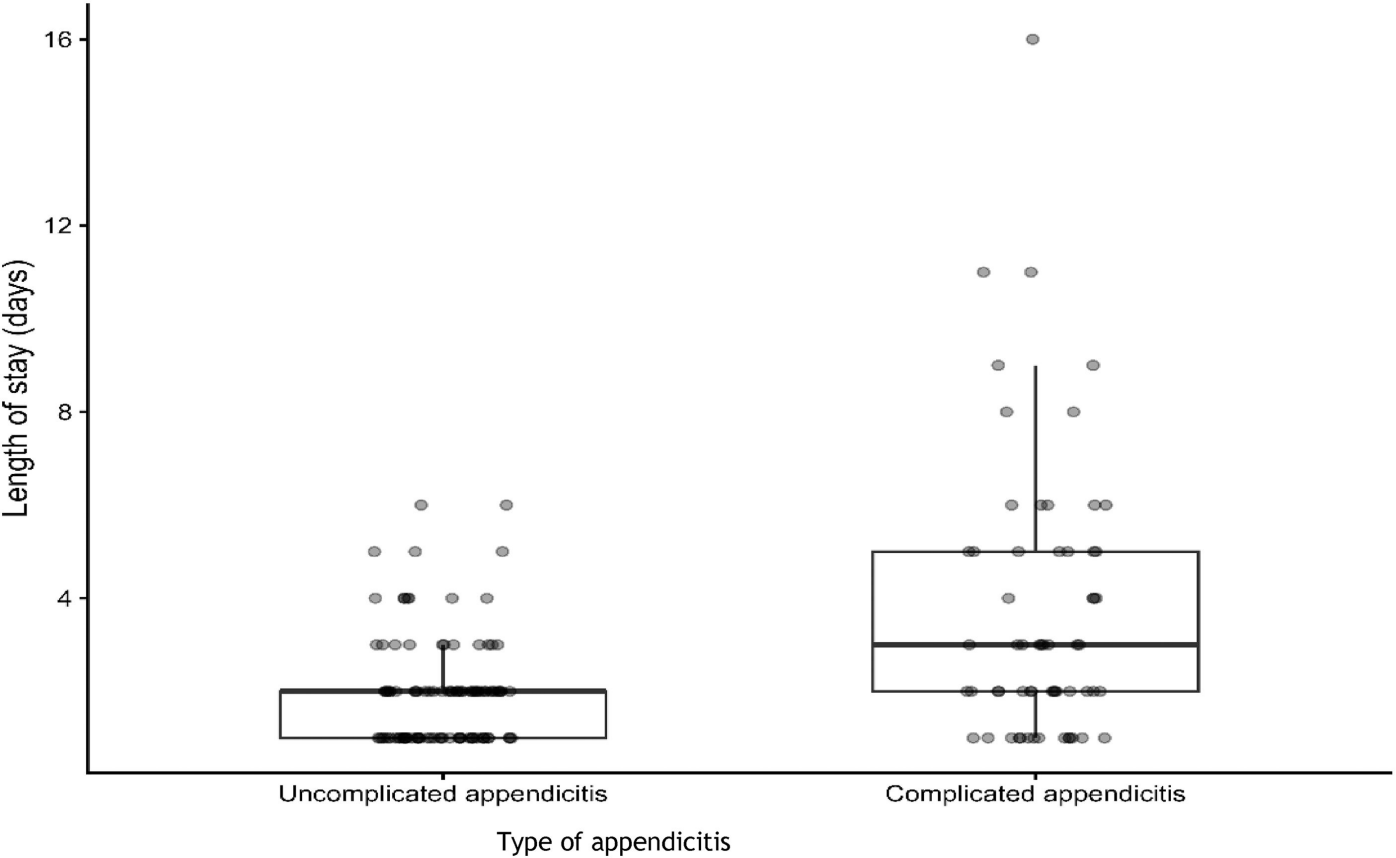
Length of hospital stay by type of appendicitis.

The median length of hospital stay was significantly longer among patients with complicated appendicitis (3.0 days [IQR: 2.0–5.0]) compared to those with uncomplicated appendicitis (2.0 days [IQR: 1.0–2.0]). (Figure 2).

Intraoperative findings, histopathological results, and postoperative outcomes are summarized in Table 3.

**Table 3:** Intraoperative characteristics, histopathological findings, and postoperative outcomes of patients with complicated and uncomplicated appendicitis.

Characteristic	Complicated appendicitis (*n* = 61)	Uncomplicated appendicitis (*n* = 107)	*p*-value
Inflamed appendix	48 (78.7)	64 (59.8)	0.013
Simple appendicitis	7 (11.5)	92 (86.0)	<0.001
Gangrenous appendix	8 (13.1)	0 (0.0)	Not applicable^†^
Perforated appendix	56 (91.8)	0 (0.0)	Not applicable^†^
Mucocele	0 (0.0)	1 (0.9)	>0.99
Phlegmon	2 (3.3)	0 (0.0)	0.13
Appendicular abscess	17 (27.9)	0 (0.0)	Not applicable^†^
Appendicular mass	2 (3.3)	0 (0.0)	0.13
Localized peritonitis	20 (32.8)	0 (0.0)	Not applicable^†^
Generalized peritonitis	8 (13.1)	0 (0.0)	Not applicable^†^
Acute appendicitis (pathology)	44 (72.1)	90 (84.1)	0.063
Suppurative appendicitis	4 (6.6)	1 (0.9)	0.060
Gangrenous appendicitis (pathology)	8 (13.1)	0 (0.0)	<0.001
Surgical site infection	4 (6.6)	1 (0.9)	0.064
Intra-abdominal abscess	6 (9.8)	1 (0.9)	0.010
Postoperative ileus	2 (3.3)	0 (0.0)	0.13
ICU admission	5 (8.2)	0 (0.0)	0.006
Hospital stay, days	3.0 (2.0–5.0)	2.0 (1.0–2.0)	<0.001

Data are presented as *n* (%) or median (IQR). Perforation, gangrene, appendicular abscess, localized peritonitis, and generalized peritonitis constitute the operational definition of complicated appendicitis and are presented primarily as descriptive characteristics of the study groups rather than independent comparative variables. *P*-values for the remaining variables were calculated using the Pearson chi-square test, Fisher's exact test, or Wilcoxon rank-sum test, as appropriate.

† These variables constitute the operational definition of complicated appendicitis and are therefore presented descriptively rather than subjected to inferential statistical comparison.

### Multivariable logistic regression analysis

In multivariable logistic regression analysis, increasing age and elevated C-reactive protein (CRP) levels were identified as independent predictors of complicated appendicitis after adjusting for potential confounders.

Each one-year increase in age was associated with a modest but statistically significant increase in the odds of complicated appendicitis (adjusted OR: 1.03; 95% CI: 1.00–1.07). Similarly, each 10 mg/L increase in CRP was associated with an 11% increase in the odds of complicated appendicitis (adjusted OR: 1.11; 95% CI: 1.05–1.17), indicating a significant association between systemic inflammation and disease severity.

Although symptom duration greater than 48 h was associated with higher odds of complicated appendicitis (adjusted OR 2.84; 95% CI 0.90 to 9.31), the association did not reach statistical significance after adjustment for potential confounding variables (*p* = 0.076).

Other variables, including sex, vomiting, fever, and white blood cell count, were not independently associated with complicated appendicitis.

Results of the multivariable logistic regression analysis are presented in Table 4.

**Table 4:** Multivariable logistic regression analysis of factors associated with complicated acute appendicitis.

Characteristic	Adjusted OR	95% CI	*p*-value
Age, years	1.03	1.00–1.07	0.046
Sex			
Male	Reference	–	–
Female	1.07	0.41–2.70	0.90
Vomiting			
No	Reference	–	–
Yes	0.59	0.24–1.44	0.30
Fever			
No	Reference	–	–
Yes	0.67	0.21–1.91	0.50
Symptom duration >48 h			
No	Reference	–	–
Yes	2.84	0.90–9.31	0.076
White blood cell count	1.07	0.98–1.17	0.12
C-reactive protein (per 10 mg/L increase)	1.11	1.05–1.17	<0.001

CI, confidence interval; OR, odds ratio.

Adjusted odds ratios were estimated using multivariable logistic regression.

## Discussion

This study provides one of the first comprehensive analyses from Somalia comparing the clinical characteristics, diagnostic scores, surgical management, and outcomes of complicated versus uncomplicated acute appendicitis. The findings demonstrate a considerable burden of complicated appendicitis, accounting for 36.3% of cases. This rate is comparable to reports from Ethiopia (36.4%) but higher than those documented in Pakistan (20%), Japan (16.1%), and India (31.8%) (8, 12–14). Furthermore, studies from South Africa report even higher proportions, with rates reaching 57% in urban settings and up to 79% in rural populations, highlighting substantial regional disparities within the African continent (15). The relatively higher proportion observed in our setting likely reflects delays in healthcare-seeking behavior, limited access to advanced diagnostic modalities, and broader systemic healthcare constraints, which contribute to disease progression prior to timely surgical intervention.

The age distribution observed in this study is consistent with global literature, with a median age of 29 years, indicating that acute appendicitis predominantly affects young adults (15–17). A male predominance was also observed, aligning with findings from international and African studies, although some regional variations have been reported (7, 16, 18–25). These patterns suggest a continued burden of disease among the economically productive population.

Right lower quadrant abdominal pain was the most common presenting feature in both groups (96.7% vs. 97.2%), consistent with classical descriptions and previous studies, including reports from South Africa where similar rates have been documented (26). Other symptoms such as vomiting, nausea, tenderness, fever, anorexia, and constipation were also frequently observed. However, as these clinical features were largely comparable between complicated and uncomplicated cases, clinical presentation alone remains insufficient to reliably distinguish disease severity, reinforcing the need for adjunctive diagnostic tools, particularly in settings characterized by delayed presentation.

Symptom duration differed significantly between groups in the unadjusted analysis, with a greater proportion of patients with complicated appendicitis presenting after 48 h (29.5% vs. 10.3%), whereas earlier presentation was more common among patients with uncomplicated appendicitis (90.7% vs. 70.5%). Overall, 17.3% of patients presented after 48 h, which is lower than rates reported in studies from Ethiopia, South Africa, and Sweden (27–30). However, after adjustment for age, sex, inflammatory markers, and other clinical variables, delayed presentation was no longer independently associated with complicated appendicitis. This finding suggests that although delayed presentation may contribute to disease progression, its apparent effect may be influenced by other patient and disease-related factors.

Imaging practices in this study reflect a combined use of ultrasound and CT scanning, with no major differences between groups. This approach is consistent with recommendations from previous studies and international guidelines (10, 31, 32). The reliance on combined imaging modalities may reflect diagnostic uncertainty or a higher proportion of patients presenting with advanced disease, emphasizing the need for context-specific diagnostic strategies in resource-limited settings.

The overall perforation rate in this study was 33.3%, occurring exclusively among patients with complicated appendicitis. This rate is higher than that reported in studies from Tanzania (25.3%), Ethiopia (26.5%), and the POSAW global study (28%), but remains within the reported range for Sub-Saharan Africa (22%–60%) (2, 16, 33–35). This finding highlights the ongoing challenge of late presentation and its impact on disease severity.

Regarding clinical scoring systems, patients with complicated appendicitis had significantly higher median Alvarado and AIR scores than those with uncomplicated appendicitis, whereas no statistically significant differences were observed for the modified Alvarado and RIPASA scores. These findings suggest an association between higher Alvarado and AIR scores and more severe disease in our cohort. However, because our analyses were limited to comparisons of score distributions between groups, we did not perform formal diagnostic performance analyses such as receiver operating characteristic (ROC) curve analysis, area under the curve (AUC), sensitivity, specificity, predictive values, or optimal cut-off value determination. Therefore, our findings should not be interpreted as evidence that one scoring system is diagnostically superior to another. Rather, they indicate that higher Alvarado and AIR scores were observed in patients with complicated appendicitis. Future prospective studies incorporating formal diagnostic accuracy analyses are needed to validate and compare the performance of these scoring systems in resource-limited settings (36–39). These findings emphasize that no single scoring system is sufficient to independently distinguish disease severity, supporting the need for an integrated diagnostic approach (36–41).

The overall postoperative complication rate in this study was 6.0%, with surgical site infection being the most common complication. This rate is higher than that reported in Ethiopia (3.8%) but lower than rates documented in other resource-limited settings such as Kenya and South Africa, where complications are frequently linked to delayed presentation (2, 29, 42). Patients with complicated appendicitis experienced worse postoperative outcomes, including higher rates of intra-abdominal abscess (9.8% vs. 0.9%) and ICU admission (8.2% vs. 0.0%), while surgical site infections were also more frequent (6.6% vs. 0.9%). These findings are consistent with the known impact of advanced disease on postoperative morbidity.

The overall average length of hospital stay was approximately 2.5 days, with longer stays observed among patients with complicated appendicitis. However, this duration was shorter than that reported in studies from Ethiopia, Kenya, Ghana, South Africa, Turkey, and Italy (2, 28, 29, 42–45). This variation may reflect differences in healthcare systems, discharge practices, and resource utilization.

Laparoscopic appendectomy was the predominant surgical approach, particularly in uncomplicated cases (95.3% vs. 73.8%), whereas open surgery (19.7% vs. 2.8%) and conversion (6.6% vs. 0.9%) were more common in complicated appendicitis. These findings are consistent with existing literature demonstrating the advantages of laparoscopic surgery, including reduced postoperative pain, shorter recovery time, lower complication rates, and improved quality of life (10, 46, 47). At the same time, the continued use of open surgery in complicated cases reflects the technical challenges associated with advanced disease, including perforation and distorted anatomy.

These findings have important clinical implications. Early risk stratification using a combination of clinical assessment, laboratory markers, clinical scoring systems, and imaging modalities may facilitate timely surgical intervention. Although reducing delays in presentation remains an important healthcare objective, our multivariable analysis suggests that age and elevated C-reactive protein were the only independent factors associated with complicated appendicitis in this cohort. Therefore, efforts to improve patient outcomes should focus on both timely access to care and accurate early identification of patients at increased risk of complicated disease.

This study has several limitations. First, its retrospective design introduces the potential for information bias, incomplete documentation, and missing data. Second, the inclusion of only surgically managed patients may have introduced selection bias and limited the generalizability of the findings. Third, the single-center nature of the study may not fully represent the broader population of patients with acute appendicitis in Somalia. Fourth, external validation of the evaluated clinical scoring systems was not performed.

In addition, several potentially important variables could not be assessed because of incomplete documentation in the medical records. Information regarding the interval from hospital admission to surgery was not consistently available and therefore could not be analyzed. Similarly, data on body mass index and comorbid conditions were incompletely recorded and could not be included in the analysis. Furthermore, important contextual factors, such as referral delays and access to healthcare services, were not fully captured. Finally, postoperative complications were not graded using the Clavien-Dindo classification because complication severity was not consistently documented in the available records.

## Conclusion

Complicated appendicitis accounted for more than one-third of acute appendicitis cases in this cohort and was associated with increased postoperative morbidity, longer hospital stay, and higher rates of ICU admission. Older age and elevated C-reactive protein levels were independently associated with complicated appendicitis. Although delayed presentation beyond 48 h was significantly associated with complicated appendicitis in the unadjusted analysis, it did not remain independently associated after adjustment for potential confounding factors. In contrast, increasing age and elevated C-reactive protein were identified as independent predictors of complicated appendicitis. Patients with complicated appendicitis had significantly higher median Alvarado and AIR scores; however, because formal diagnostic performance analyses, including ROC curve analysis, AUC estimation, sensitivity, specificity, predictive values, and optimal cut-off value determination, were not performed, no conclusions regarding the comparative diagnostic performance of these scoring systems can be drawn. Prospective multicenter studies incorporating formal diagnostic accuracy analyses are warranted to validate these findings and optimize risk stratification in resource-limited settings.

## Data Availability

The original contributions presented in the study are included in the article/Supplementary Material, further inquiries can be directed to the corresponding author.
